# Rare Case of First Permanent Molar Primary Failure of Eruption with Agenesis of Premolars

**DOI:** 10.3390/children11020152

**Published:** 2024-01-25

**Authors:** Arina Vinereanu, Aneta Munteanu, Francois Clauss, Eusebiu Vlad Gorduza

**Affiliations:** 1 Faculty of Dentistry, Carol Davila University of Medicine and Pharmacy, 050474 Bucharest, Romania; arina.vinereanu@umfcd.ro; 2 Faculty of Dental Surgery, University of Strasbourg, 67081 Strasbourg, France; francois.clauss@chru-strasbourg.fr; 3 Faculty of Medicine, Grigore. T. Popa University of Medicine and Pharmacy Iași, 700115 Iași, Romania; eusebiu.gorduza@umfiasi.ro

**Keywords:** primary failure of eruption, premolar agenesis, case report

## Abstract

Primary failure of eruption (PFE) is a rare non-syndromic condition involving total or partial non-eruption of posterior teeth in the absence of mechanical obstruction. This paper presents the case of a healthy girl referred at age 4 for asymmetry of the upper arch. Clinical examination confirmed a slight shift of the upper midline to the left, with no erupted teeth distal to the upper left canine and a left posterior open bite. Panoramic X-rays showed delayed intraosseous development of the lateral left upper teeth compared to the right side. Clinic and radiographic follow-up during the next 9 years showed that 26 had not erupted by almost 13 years of age, 27 had delayed development and an unusual shape, and there was an agenesis in 24 and 25. Genetic analysis using the *PTH1R* single-gene sequencing method did not detect any known disease-causing or rare pathogenic variants that could explain the patient’s phenotype. Even when detected early, PFE raises difficult problems with regard to diagnosis and ortho-surgical treatment planning due to the impossibility of accurately predicting its evolution. Tooth agenesis on the same arch worsens the prognosis and adds challenges to planning the treatment. Close long-term follow-up and timely adjustment of the treatment plan in accordance with the evolution of the case are needed.

## 1. Introduction

Tooth eruption is a unique, genetically determined process [1]. Variations and abnormalities in tooth eruption may occur both in syndromic and non-syndromic patients and may cause serious orthodontic disorders. Alterations of the eruption process in terms of timing, sequence, and extent need to be noticed, monitored, and adequately managed in order to minimize potential unwanted consequences [2].

Various changes in the eruption process have been described, with a range of causes, either general (endocrine, carential, systemic, and drug-induced) or genetic [3]. Primary failure of eruption (PFE) is defined as a non-syndromic condition involving total or partial non-eruption of posterior teeth in the absence of mechanical obstruction [4,5]. It is a rare disease with a reported prevalence of 0.06% [6]. PFE may affect deciduous and/or permanent teeth [2,5]. Involved teeth are either completely retained or can erupt partially and then cease to erupt, remaining relatively submerged. As only lateral teeth are affected, the posterior open bite is the characteristic clinical feature, with functional impairment as a consequence [7,8]. The disorder may occur in any or all of the posterior quadrants, and it is rarely symmetric [4,7]. All teeth situated distal to the most mesial affected tooth are typically involved [4,7].

Clinical features and severity degrees may vary widely in non-syndromic PFE [8]. A systematic review performed by Hanisch et al. (2018) on previous published studies (2006–2017) on PFE summarized that girls tend to be slightly more affected (1.38:1 ratio), in almost 70% of the reported cases both molars and premolars were involved, primary teeth were affected in only about ¼ of the situations, while in most cases (75.7%) PFE was limited to permanent teeth and the condition was more frequently bilateral (64%) [5].

The etiology of PFE is still subject to study. Associations between eruption failure and other dental anomalies of known genetic origin suggest that PFE may have a significant genetic component [9]. Alterations in two genes have been associated with PFE so far. Mutations in parathyroid hormone receptor 1 (*PTH1R*) have been identified in several familial cases of PFE [2,8,10]. Decker et al. (2008) first reported that a variant in *PTH1R* was associated with PFE. This gene is located on chromosome 3p21-p22.1, MIM #, and contains 16 exons [11]. According to Roth et al. (2014), mutations in *PTH1R* are associated with lethal dwarfism, chondrodysplasia, and isolated dental disorders specific to eruption [12]. More recently, reported mutations in the lysine methyltransferase 2C *(KMT2C)* gene have been suggested as another potential molecular etiology of familial non-syndromic PFE [13]. However, not all patients with PFE carry mutations in known genes [1,14].

The diagnosis of certainty in PFE is a difficult task. Checking parents’ occlusion is the first step recommended by Yamaguchi et al. (2022), as in most cases there is a family history [15]. This should be followed by the exclusion of any local and systemic causes for a potential mechanical impairment of the eruption. Ankyloses, cysts, interference of adjacent teeth, lateral pressure from the tongue, and oral clefts are cited as local factors susceptible to causing eruption failure, while genetic syndromes, endocrine disorders, and long-term chemotherapy are mentioned as systemic factors [10,15]. More than 40 genetic diseases or syndromes are associated with failure of eruption, e.g., cleidocranial dysplasia, osteopetrosis, Rutherford syndrome, GAPO syndrome, and osteoglophonic dysplasia [9,15]. Infraocclusion, immobility, metallic sound on percussion, and radiographic obliteration of the periodontal ligament space are cited as indicators for mechanical failure of eruption (ankylosis) [15]. In practice, however, it may actually be very difficult to accurately differentiate between PFE and ankylosis. Therefore, genetic tests looking for variations in the *PTH1R*, whenever possible, may help differential diagnosis [10,11].

Treatment of PFE can be very challenging, as the affected teeth do not react to orthodontic forces, regardless of the type or clinical severity of the condition [5,16]. This suggests that the defect in the eruptive process is permanent and irreversible. The involved teeth are not only unresponsive to orthodontic mechanics but tend to become ankylosed when orthodontic traction is applied [4,15,17]. Orthodontic traction is therefore not to be attempted when the diagnosis of PFE is confirmed [4,10,15,18]. Treatment of PFE requires an interdisciplinary approach [8,18]. Each case needs to be carefully evaluated, and treatment planning must be carried out in accordance with the clinical situation and the patient’s age [5]. In mild cases, with a limited number of insufficiently erupted teeth, prosthodontic restoration may be enough to establish a satisfactory occlusion. More severe situations may require a surgical approach of variable complexity, from small-segment osteotomy with or without bone grafting [4,19] to extraction of affected teeth followed by ridge augmentation and implant placement [15]. In very severe cases, with a large number of affected teeth and a significantly deformed alveolar ridge, removable appliances may be considered in order to restore occlusion and appearance from the early stages [20].

The aim of the study was to bring to attention the evolution of a case of PFE followed since primary dentition.

## 2. Case Report

A 4-year-old girl was presented in a private clinic in Bucharest for asymmetry of the upper arch. The patient had no systemic disorder; her stature was normal for her age. There was no history of trauma or surgical events. The patient’s mother had undergone orthodontic treatment for crowding during her childhood, and both parents had full dental arches. Anamnesis revealed no other relevant family history of dental abnormalities.

**Figure 1:**
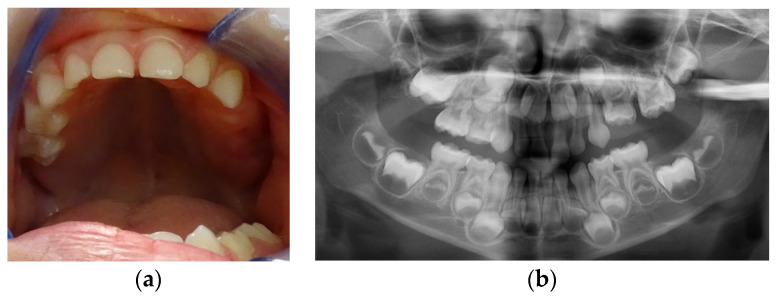
First visit; age of 4 y and 4 m. (**a**) Clinical aspect of the upper arch: no erupted teeth distal to the left upper primary canine; (**b**) panoramic X-ray.

Clinical examination (Figure 1a) revealed no erupted teeth distal to the left upper canine, a left lateral open bite, and a slight shift of the upper midline to the left. Panoramic X-rays taken at this time showed delayed intraosseous development of the lateral left upper teeth as compared to the right side (Figure 1b) and no sign of mechanical obstruction. The agenesis of upper left premolars (24 and 25) was suspected but uncertain at the time.

**Figure 2:**
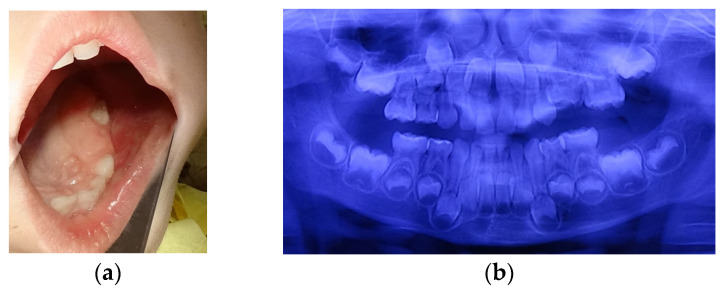
Age of 5 y and 3 m. (**a**) Occlusal view of the left upper arch; (**b**) panoramic X-ray.

At the age of 5 y and 3 m, a molar erupted in the left upper arch. The erupting tooth was considered to be the second primary molar (65), although its shape and size, as seen on the new panoramic X-ray, substantially differed from those of 55, present on the arch. This new X-ray also suggested that 64 could be mechanically impacted by 63 (Figure 2a,b).

**Figure 3:**
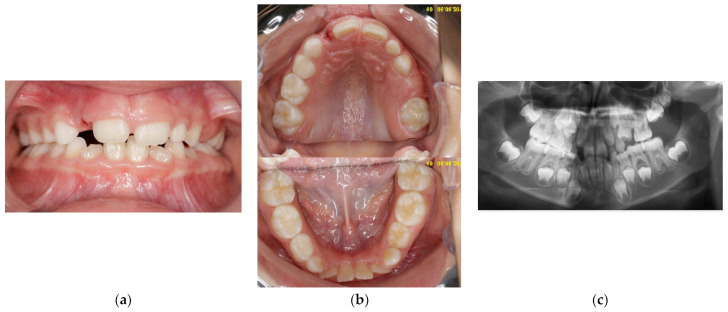
Age of 7 y and 10 m. (**a**) Frontal view showing slight shift of the upper midline to the left and lower crowding; (**b**) occlusal view of both arches: 65 fully erupted, with atypical crown shape and size; (**c**) panoramic X-ray showing impacted 64, bimaxillary crowding, and a tendency for re-inclusion of 74.

At the age of 7 y and 10 m, 65 had erupted, with an atypical crown shape and size (larger than usual, almost like a first permanent molar) (Figure 3a,b). Furthermore, no premolar buds could be seen in the upper left quadrant, and the unerupted 26 had reached the coronal stage, while 27 had a delayed development as compared to 17 (Figure 3c). Tooth 64 had become more intruded and seemed to be impacted by 63. PFE of the upper left permanent molars was suspected. Bimaxillary crowding and class II malocclusion were also noted. Caries prevention measures were applied, such as professional cleaning and glass-ionomer sealants on erupted first permanent molars (16, 36, and 46).

**Figure 4:**
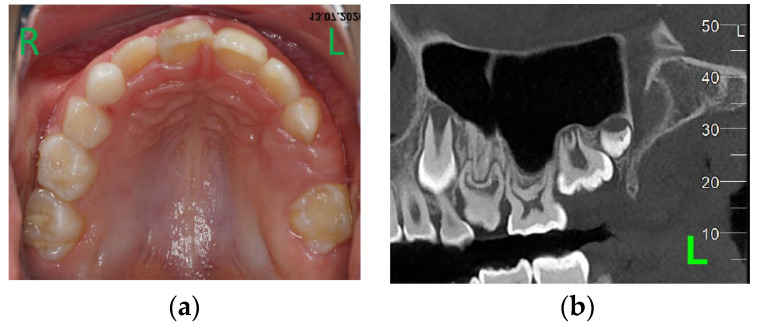
Age of 9 y and 7 m. (**a**) Occlusal view of the upper arch; (**b**) CBCT confirmed agenesis of 24, 25, and impaction of 64.

At the age of 9 y and 7 m, 26 was still not on the arch (Figure 4a). A cone beam computed tomography (CBCT) examination was performed. Agenesis of 24 and 25 became certain (Figure 4b), and no areas of ankylose in teeth with eruption failure were found. A decision was taken to remove 63 in order to favor the eruption of mechanically impacted 64, but extraction was postponed at the family’s request.

**Figure 5:**
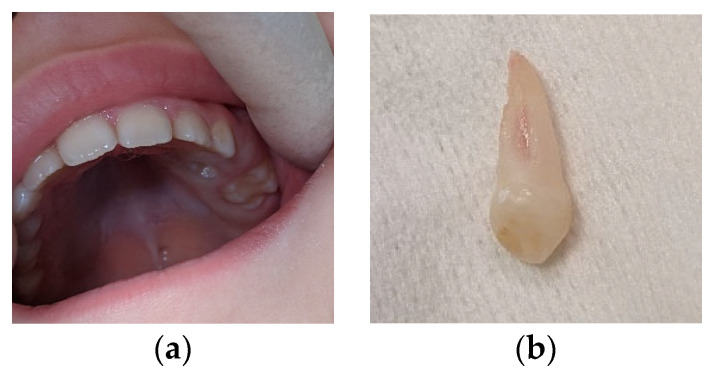
Age of 10 y and 8 m. (**a**) Clinical view, with impacted 64; (**b**) 63 extracted.

At the age of 10 y and 8 m, 63 was extracted (Figure 5), giving way for 64, while 65 had reached the occlusion plane and was sealed with a glass-ionomer-based sealant.

Clinical examination at the age of 10 y and 11 m revealed that 74 became more severely re-intruded. The tooth was removed under local anesthesia.

**Figure 6:**
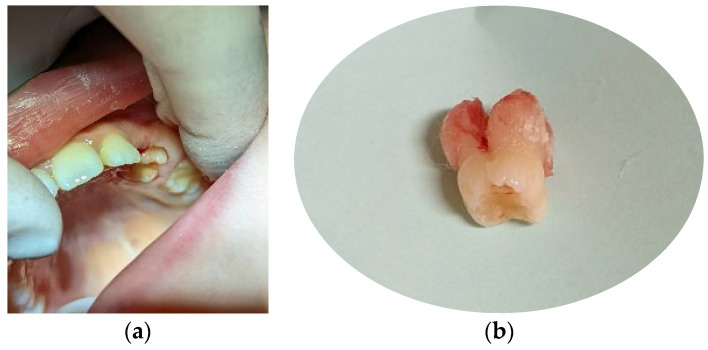
Age of 11 y and 3 m. (**a**) Clinical aspect of emerging 64; (**b**) 64 extracted before full eruption.

Erupting 64 was subsequently removed at the age of 11 y and 3 m under nitrous sedation (Figure 6) in order to favor eruption of the upper left canine. Unusual crown and root shapes of 64 were noted (Figure 6b).

**Figure 7:**
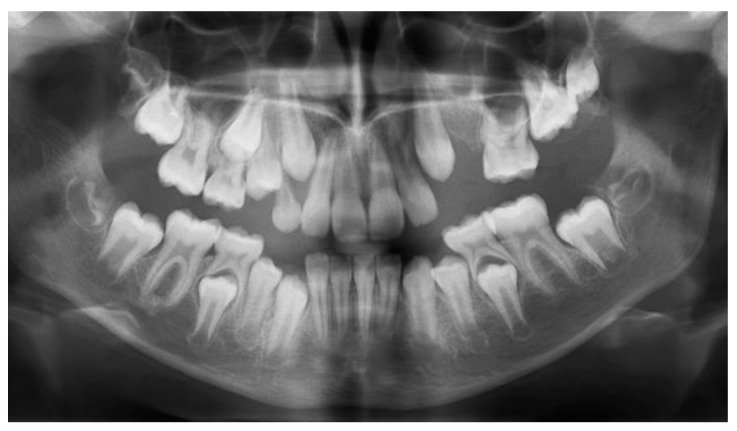
Panoramic X-ray at the age of 11 y and 8 m showed delayed development of 26 and 27.

At the age of 11 y and 8 m, 23 was gaining its place on the arch (simultaneously with 13), 65 remained stable, and 26 and 27 seemed to continue their development very slowly within the alveolar bone (Figure 7).

Genetic testing was proposed in order to identify potential underlying genetic factors for the diagnosis of PFE. After considering the pros (adding data for diagnostic purposes) and cons (relatively high costs and not supported by the national health trust), the patient’s parents decided not to do genetic analysis at this time.

It is important to summarize that, given the impossibility of accurately foreseeing the evolution in the upper left quadrant, a wait-and-watch strategy was chosen, keeping therapeutic interventions to a minimum during the whole follow-up period. In addition to routine caries prevention measures (sealants and professional cleaning), treatment mainly consisted of surgically removing deciduous teeth unable to spontaneously exfoliate in order to favor the best possible development of the situation under the given circumstances.

Taking into account the presence of third molars’ buds in the upper right quadrant and in the mandible, bimaxillary crowding, and the missing upper left premolars, a treatment plan was made that involved keeping 65, the removal of 14, 34, and 44 in order to favor a balanced occlusion and orthodontic correction of the midline, while waiting and observing the evolution of the upper left permanent molars. As previous case reports found orthodontic attempts to bring retained teeth onto the arch unsuccessful, for the time being, this was not considered an option for 26 and 27. The treatment plan was explained to the child and parents. Extraction of permanent teeth was regarded as a major drawback by the patient’s family, while displacement of the midline remained the main concern. Any further action was postponed, and the case remained under observation.

**Figure 8:**
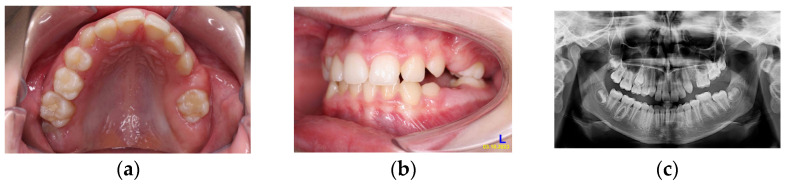
Age of 12 y and 11 m. Intraoral pictures (**a**,**b**) and panoramic X-rays (**c**) showed retained 26 and 27.

At the age of 12 y and 11 m, while the upper canines were erupting, the clinical and radiological situation in the distal part of the upper left quadrant was not much different from before (Figure 8a–c).

At this point, the decision was taken by parents to perform genetic testing. Sequence and Del/Dup (CNV) analysis using the Blueprint Genetics (BpG) *PTH1R* single-gene test did not detect any known disease-causing or rare variants that could explain the patient’s phenotype as described to the laboratory at the time of interpretation.

The case remains under clinical surveillance.

## 3. Discussion

Proffit and Vig first introduced the term “primary failure of eruption” (PFE) to describe a condition in which a troubled eruption mechanism causes non-ankylosed teeth to be unable to erupt [4].

Raghoebar et al. (1991) proposed two different terms for localized eruption failure: primary retention (1) defines those situations where eruption is arrested before the crown has penetrated the oral mucosa, while secondary retention (2) stands for those cases where the tooth has penetrated the oral mucosa but is not able to finalize the eruption process [21,22]. By this classification, our patient seems to be a primary retention case, with the first and second upper left permanent molars unable to emerge. Frazier-Bowers et al. (2007) divided PFE into three different types. In Type I, all affected teeth show a similar or severe lack of eruption potential. In Type II, the teeth situated distally from the most mesial-affected tooth show greater yet still inadequate eruption potential. Subjects displaying both Type I and II PFE are diagnosed with Type III [7]. According to this classification, our patient fits into the Type I pattern.

All clinical features described in the presented patient suggest a diagnosis of PFE involving permanent upper left molars, co-existing with agenesis of both upper left premolars and unusual shapes and dimensions of upper left primary molars, particularly the crown of 65 and roots of 64. No one in her family had a history of eruption failure. According to Hanish et al., almost 15% of patients with PFE had no family members affected by PFE [5]. It is considered that the patient with PFE without family history may have had a spontaneous mutation in *PTH1R* [14]. This hypothesis was not confirmed in our patient, as genetic testing performed at almost 13 years of age found no mutations in the *PTH1R* gene. No further genetic testing was performed.

Except for the lack of genetic confirmation, all other factors indicate the diagnosis of PFE in our patient. Although mechanical impaction of 64 could not be ruled out and was clinically managed until the tooth erupted and could be removed, syndromic and local factors that might have caused eruption failure in the permanent molars were excluded, as recommended by Yamaguchi et al. (2022) [10] and Frazier-Bowers et al. (2010) [14].

The unusual crown shape and size of 65 are to be noted, along with the unusual root anatomy of the extracted, previously impacted 64. Abnormalities noticed in our patient (alteration of crown shape and size/root morphology, mechanical impaction, hypodontia) are found among those reported in other studies. Hanish et al. (2016) performed a systematic review of 17 articles reporting clinical data on 314 patients with PFE. In 39 cases, additional dental anomalies were described. These included alterations in the root morphology (*n* = 11), impacted teeth (*n* = 10), delayed eruption of further teeth (*n* = 6), hypodontia (*n* = 5), hyperdontia (*n* = 3), transposition of teeth (*n* = 2), peg-shaped teeth (*n* = 1), and mechanical failure of eruption (*n* = 1). In 70 cases, no further dental anomalies were reported, while no details regarding these data were available for 190 cases [5].

Regular clinic and radiographic follow-up were performed during the next 9 years in order to monitor the eruption sequence and occlusion during growth. CBCT performed at age 9 ascertained the agenesis of premolars in the same quadrant where delayed evolution of both deciduous and permanent molars was seen. Ahmad et al. [9] reported that 13% of the patients with PFE had hypodontia, a percentage substantially higher than within the regular population [23].

Regarding the type of malocclusion associated with PFE, no consensus exists in the literature. Hanisch et al. (2018) report 5.9% of skeletal Classes I, 14.7% of Classes II, and 79.4% of Classes III [5]. Yamaguchi et al. (2011) and Awad et al. (2022) cited the predominance of skeletal Classes III [1,24]. Our patient had a Class II malocclusion.

In our case, the patient was a healthy 4-year-old girl who initially presented with a shortened left upper arch due to the delayed eruption of the deciduous molars. She was closely monitored during the following 9 years and remains under clinical surveillance. It is important to mention that, to the authors’ knowledge, there are no reports on PFE cases described and monitored since such a young age and for such a long period of time. A systematic review performed on 314 patients with PFE, aged between 8 and 58 years, showed that in only 24.3% of cases, primary teeth were affected [5]. The present case was initially diagnosed as a unilateral PFE during the primary dentition (age of 4 y). However, during follow-up, 65 had fully erupted by age 7 y 10 m, while 64 was mechanically impacted and managed to partially erupt after removal of the deciduous canine. We can therefore consider that, in our case, primary teeth were not affected by PFE but rather by a delayed eruption.

The described case bears some of the features described in previous reports of PFE, such as tooth agenesis, eruption delay, impacted teeth, tooth shape and size anomalies, and asymmetrical arches. Our patient was followed from a very young age, with deciduous dentition, and valuable information on the case evolution was gathered over these 9 years. Literature points out that treatment planning in PFE is particularly challenging due to difficulties in diagnosis as well as the impossibility of accurately predicting the evolution of non-erupted teeth, and realistic treatment goals should be pursued rather than ideal results. The age of our patient added challenges to managing the case. Having a rare condition has an important psychological impact. In addition to that, it was not easy for such a young child to accept several teeth being surgically removed, especially when compared to peers who never had to face similar situations. Although timely extraction of three premolars could have helped balance the occlusion and correct the midline with minimal orthodontic intervention, patient and family reluctance is understandable, and the patient’s perspective is, in every case, a very important factor to take into account. Treatment of PFE can only be finalized after the end of the growth process.

This case report points out the importance of long-term monitoring of PFE cases, whether genetically confirmed or not. The treatment plan needs to be carefully adjusted over time, in accordance with the evolution of the case. Further research is needed in order to clarify the etiology of PFE, potential links between the genetic substrate and specific features, and efficient treatment solutions with a higher degree of predictability.

A limitation of this case report is the fact that, despite the long-term follow-up, treatment is not completed and needs to be extended, with a multidisciplinary approach, for a long period of time. The fact that only one patient is described, with clinical, radiological, and genetic findings, gives this study a low level of evidence.

Underlying genetics in PFE is still unclear; not all patients with PFE carry mutations in known genes [1,14]. In the presented case, 26 has not erupted by almost 13 years of age, and 27 has delayed development (and an unusual shape). These findings, along with the absence of the premolars and the unusual shape and size of the deciduous molars, as well as the patient’s gender, made us consider that PFE is the most likely diagnostic, possibly falling into another, perhaps more rare, genetic category. The etiology of PFE is yet to be clarified by research, as are the potential links between genetic alterations, clinical features, and the evolution of affected teeth.

## 4. Conclusions

Even when detected early, PFE raises difficult problems with regard to diagnosis and treatment planning due to the impossibility of accurately predicting its evolution. Tooth agenesis on the same arch worsens the prognosis and adds challenges to planning the treatment. Close long-term follow-up and timely adjustment of the treatment plan in accordance with the evolution of the case are needed, while the patient’s and family’s expectations need to be carefully considered at all stages.

## Data Availability

The data presented in this study are available on request from the corresponding author. The data are not publicly available due to patient privacy reasons.
